# Differential kinetics of splenic CD169+ macrophage death is one underlying cause of virus infection fate regulation

**DOI:** 10.1038/s41419-023-06374-y

**Published:** 2023-12-18

**Authors:** Valentina Casella, Eva Domenjo-Vila, Anna Esteve-Codina, Mireia Pedragosa, Paula Cebollada Rica, Enric Vidal, Ivan de la Rubia, Cristina López-Rodríguez, Gennady Bocharov, Jordi Argilaguet, Andreas Meyerhans

**Affiliations:** 1 Infection Biology Laboratory, Department of Medicine and Life Sciences (MELIS), Universitat Pompeu Fabra, 08003 Barcelona, Spain; 2 CNAG-CRG, Centre for Genomic Regulation (CRG), Barcelona Institute of Science and Technology, 08028 Barcelona, Spain; 3 Universitat Pompeu Fabra (UPF), 08003 Barcelona, Spain; 4 Unitat mixta d’Investigació IRTA-UAB en Sanitat Animal. Centre de Recerca en Sanitat Animal (CReSA), Campus de la Universitat Autònoma de Barcelona (UAB), 08193 Bellaterra, Catalonia Spain; 5 IRTA, Programa de Sanitat Animal, Centre de Recerca en Sanitat Animal (CReSA), Campus de la Universitat Autònoma de Barcelona (UAB), 08193 Bellaterra, Catalonia Spain; 6 EMBL Australia Partner Laboratory Network at the Australian National University, Acton, Canberra, ACT 2601 Australia; 7 Immunology Unit, Department of Medicine and Life Sciences (MELIS), Universitat Pompeu Fabra, 08003 Barcelona, Spain; 8 Marchuk Institute of Numerical Mathematics, Russian Academy of Sciences, 119333 Moscow, Russia; 9 Sechenov First Moscow State Medical University, 119991 Moscow, Russia; 10 Institució Catalana de Recerca i Estudis Avançats (ICREA), 08010 Barcelona, Spain

**Keywords:** Viral infection, Cell death and immune response, Immune cell death, Imaging the immune system

## Abstract

Acute infection and chronic infection are the two most common fates of pathogenic virus infections. While several factors that contribute to these fates are described, the critical control points and the mechanisms that underlie infection fate regulation are incompletely understood. Using the acute and chronic lymphocytic choriomeningitis virus (LCMV) infection model of mice, we find that the early dynamic pattern of the IFN-I response is a differentiating trait between both infection fates. Acute-infected mice generate a 2-wave IFN-I response while chronic-infected mice generate only a 1-wave response. The underlying cause is a temporal difference in CD8 T cell-mediated killing of splenic marginal zone CD169+ macrophages. It occurs later in acute infection and thus enables CD169+ marginal zone macrophages to produce the 2nd IFN-I wave. This is required for subsequent immune events including induction of inflammatory macrophages, generation of effector CD8+ T cells and virus clearance. Importantly, these benefits come at a cost for the host in the form of spleen fibrosis. Due to an earlier marginal zone destruction, these ordered immune events are deregulated in chronic infection. Our findings demonstrate the critical importance of kinetically well-coordinated sequential immune events for acute infection control and highlights that it may come at a cost for the host organism.

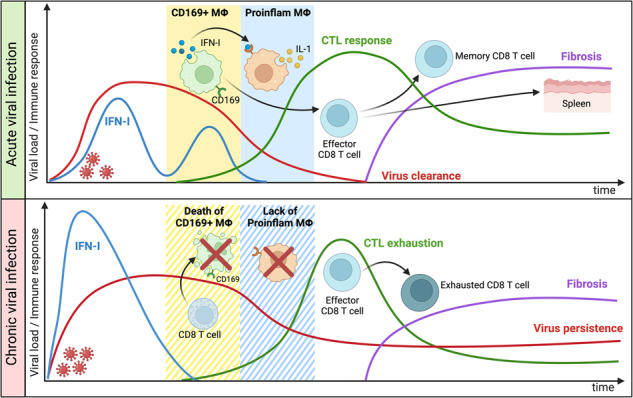

## Introduction

The fate of a virus infection is determined by the dynamic interplay between the expanding virus and the concomitantly induced immune response. It can be categorized as “acute” or “chronic” depending on the temporal virus-host relationship [1]. In humans, acute infections are commonly resolved within a few weeks. Once the infection is under control, tissue damage has to be repaired and immune homeostasis re-established. In contrast, chronic infections are not resolved and emerge when innate and adaptive immune responses are not sufficient to eliminate the invading virus during the primary infection phase. In this case, the host immune response has to adapt to the continuous presence of the invading virus and downregulate immune effector mechanisms to avoid immunopathology. This is achieved by activating suppressive activities including T cell exhaustion [2–5], the generation of monocyte-derived suppressor cells (MDSCs) [6, 7] and regulatory cell subsets [8].

Virus and host factors contribute to infection fate decisions and physiological infection outcomes. Virus factors include host cell tropism, infection dose, virus immune evasion mechanisms and the capacity to latently infect cells [9, 10]. From the host, it is age, genetic make-up and immune competence that affect how a virus infection evolves [11, 12]. Conceptually, infection outcomes are based on the two simple principles “numbers game” and “immune geography” [13]. These terms refer to the kinetics of virus growth and immune reactions, and the spatial organization of the immune response in relation to viral entry sites and tissue tropism [14]. Together they determine cytokine gradients and immune cell homing patterns across tissues that ultimately regulate infection fates and pathogenicity [15–17]. However, while both principles provide a rationale for a dynamic view on virus infection regulation, they do not specify when and how a particular infection fate is mechanistically determined. Here we use the LCMV-Doc mouse model of acute and chronic virus infection to characterize the spatiotemporal events underlying both infection courses. We show that the decisive event for the infection fates is the different destruction kinetics of CD169+ marginal zone macrophages. These cells are key coordinators of adaptive immune responses. Their presence is required during a specific time window and ensures the efficient virus control in an acute infection. Together our results highlight the critical importance of kinetic coordination of innate and adaptive effector mechanisms for pathogen control.

## Results

### Differential kinetics of type I IFN genes during acute and chronic LCMV infections

**Fig. 1:**
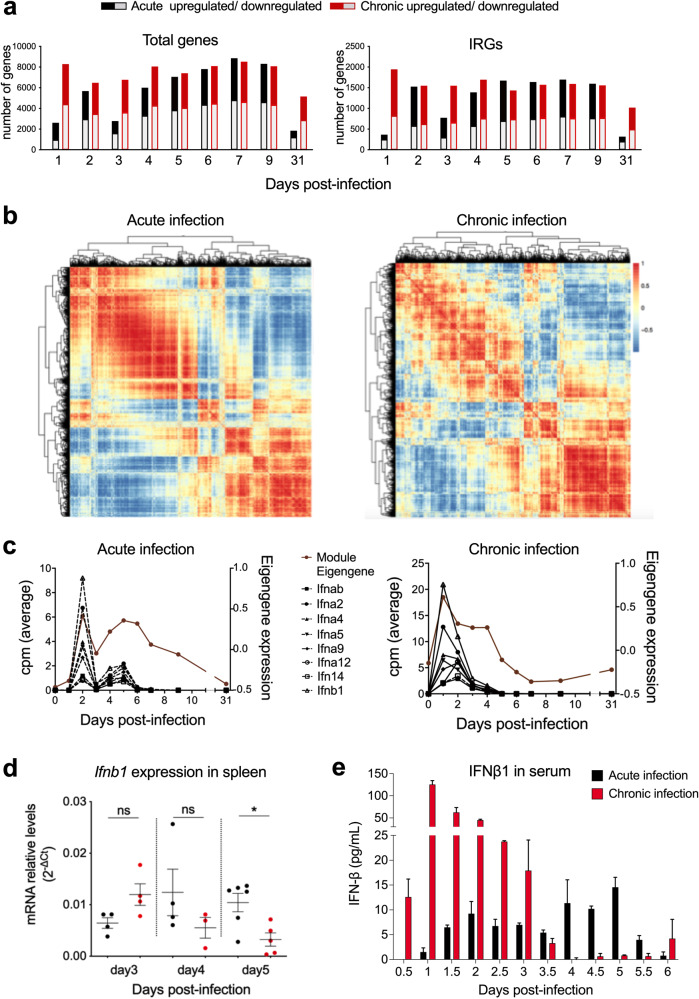
Differential type I IFN responses in acute and chronic LCMV infections. **a** Numbers of upregulated and downregulated total and interferon-regulated genes that were differentially expressed in spleens at the indicated time points after acute and chronic LCMV infections. The transcriptome of naive mice is used as reference. **b** Pearson’s pairwise correlation matrix showing IRGs expression patterns in acute and chronic infections. The color scale indicates the degree of correlation. **c** Eigengene expression profiles from acute-brown and chronic-brown modules are represented (brown lines) together with expression kinetics of IFN-I genes contained in the same modules (black lines). **d** qPCR of *Ifnb1* from spleens of acute- (black) and chronic-(red)infected mice at the indicated time points post-infection. The relative gene expression level was normalized to *Gapdh*. **e** ELISA of IFNB1 from serum of acute- and chronic-infected mice at the indicated time points post-infection. For each group and time point the mean ± SEM is shown. *p ≤ 0.05; unpaired two-tailed t-test.

To identify early differentiating traits of acute and chronic virus infections, we used the well-established lymphocytic choriomeningitis virus (LCMV) mice infection model. It is the “gold-standard” for the analysis of interactions between a non-cytopathic virus with its host organism and enables the establishment of acute and chronic infections in two ways [18]: (i) the use of high dose infections with the virus strains LCMV-Arm and LCMV-Cl13 that differ in their replication rate and target cell infectivity [19], and (ii) low or high dose infection with LCMV-Doc [20]. Since we were interested in differentiating traits of the host responses, we chose the latter option that excludes differences in viral properties. C57BL/6J mice were infected with a low-dose (2 ×10^2^ plaque-forming units (PFU)) or a high-dose (2 ×10^6^ PFU) of LCMV-Doc to establish an acute or chronic infection, respectively. A detailed description of virus kinetics and T cell responses during both infections has been given previously [21, 22]. To identify early global differences between both infection fates, time-resolved splenic transcriptomes were determined by RNAseq every 24 h from day 0 to day 7, and at days 9 and 31 post-infection (p.i.). We previously used a similar approach to determine differences in late host responses [21]. Taking non-infected mice as a reference point, 15919 differentially expressed genes between acute and chronic infections were identified (Fig. 1a, left panel). To narrow down the analyses to early antiviral innate immune responses, the differentially expressed genes that were regulated by type I interferons (IFN-I) were selected through the interferome database (http://www.interferome.org). This led to 2825 differentially expressed interferon-regulated genes (IRGs; Fig. 1a, right panel). The number of differentially expressed total genes and IRGs during chronic infection remained roughly constant over time, reaching maximum levels already at d1 p.i. In contrast, transcriptomic changes in acute LCMV-infected mice showed a biphasic dynamics characterized by an initial peak at d2 p.i., followed by a gradual increase reaching the same level as in chronic infection by d7 p.i. (Fig. 1a). To further characterize the IFN-I response behavior in acute and chronic infections, a correlation matrix of the differentially expressed IRGs was generated (Fig. 1b). IRGs showed well-defined clusters of co-expression during acute infection while displayed a more heterogeneous pattern during chronic infection, indicating a differential regulation of IFN-I responses during both infection fates.

To explore splenic transcriptome dynamics in acute and chronic infections, two signed coexpression networks were constructed from all differentially expressed genes via weighted gene correlation network analysis (WGCNA) [23]. This methodology allows to cluster genes with similar patterns of expression and enables the definition of modules of highly coexpressed genes (Supplementary Fig. S1a) [23]. Twenty and twenty-one modules from acute and chronic infections were identified, respectively, and color-coded for identification purposes (Supplementary Fig. S1b). Two modules, the acute-brown module of acute infection and chronic-brown of chronic infection, were enriched in the gene ontology (GO) term “cellular response to interferon-beta” (Supplementary Fig. S2). These two modules contained *Ifnb1* and seven *Ifna* genes (Fig. 1c) together with several IRGs (Supplementary Fig. S3a). Importantly, the module eigengenes (Fig. 1c, brown lines) and the individual IFN-I genes (Fig. 1c, black lines) showed different expression kinetics in acute and chronic infections. Acute infected mice showed two IFN-I gene expression peaks at d2 and d5 p.i., while chronically-infected mice had only a single peak at d1 p.i.

The first wave of IFN-I expression in acute and chronic infection is known to be produced from plasmacytoid dendritic cells (pDCs) [24], however the second IFN-I expression wave as a differentiating trait between both infection fates has not been described. To validate the second wave of IFN-I at the transcript and protein level, we quantified the transcripts of *Ifnb1* (Fig. 1d) and of the IRG *Mx1* (Supplementary Fig. S3b) in spleens from additional infected mice by quantitative PCR (qPCR) at days 3, 4 and 5 p.i., and the IFNB1 protein in serum every 12 h from day 0 to day 6 post acute and post chronic infection by an IFNB ELISA (Fig. 1e). Overall, transcript and protein data are in good agreement. Splenic transcript and serum IFNB1 protein levels peak at day 2 and day 1 in acute and chronic infection, respectively. Peak IFNB1 levels are about 15-fold higher in chronic infection, similar to what has been previously described [25]. While IFNB1 then continuously declines in chronic infection, a second IFNB1 wave is observed in acute infection from days 3.5 to 6 (Fig. 1e). Since these early differences in the IFN-I response may contribute to the regulation of the infection fate, we subsequently focused on the characterization of the second wave of IFN-I and its downstream effects.

### CD169+ marginal zone macrophages produce the second wave of IFN-I expression during acute LCMV infection

**Fig. 2:**
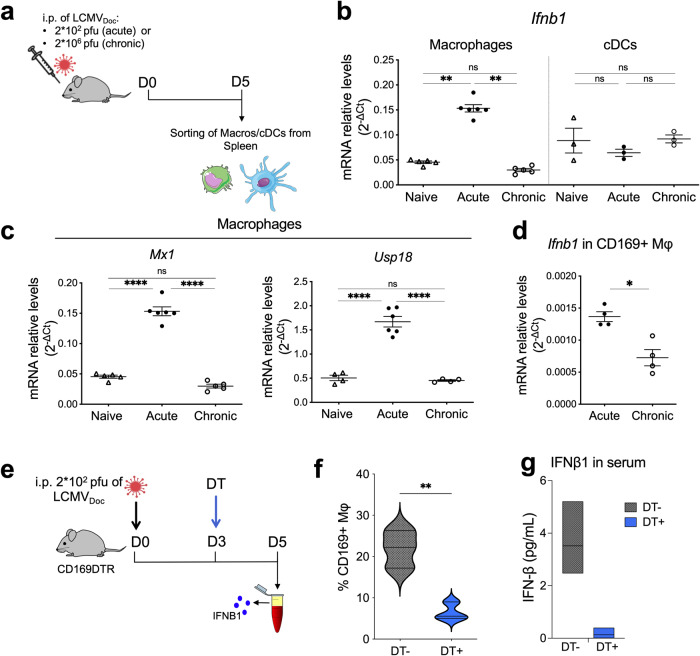
Analysis of type I IFN response at day 5 post- acute and -chronic LCMV infections. **a** Schematic representation of LCMV infections and cell sorting. **b** *Ifnb1* expression levels measured by qPCR using RNA extracted from sorted macrophages (macros) and conventional dendritic cells (cDCs) of naive or infected mice. **c** qPCR of *Mx1* and *Usp18* from macrophages sorted from acute- and chronic-infected mice. **d** *Ifnb1* expression levels in splenic CD169+ macrophages obtained at day 5 p.i. To obtain sufficient number of cells mice were previously injected in vivo with a biotin-labeled anti-CD169 antibody, and CD169+ macrophages cells were isolated ex vivo by magnetic separation using anti-biotin microbeads, and further purified by sorting after staining with Streptavidin-Phycoerythrin. The relative gene expression levels were normalized to *Gapdh*. **e** Schematic representation of diphtheria toxin (DT) treatment in acutely LCMV-infected CD169DTR mice. Flow cytometry quantification of splenic CD169+ macrophages (**f**) and ELISA quantification of IFNB1 in serum (**g**) from CD169DTR mice untreated (DT−) or treated (DT+) with diphtheria toxin (DT), at day 5 post-infection. For each group the mean ± SEM is shown; significance is determined via one-way ANOVA or unpaired two-tailed t-test; ns not significant; *p ≤ 0.05; **p ≤ 0.01; ****p ≤ 0.0001.

Acute and chronic LCMV infections lead to an early depletion of pDCs and a shift of the main IFN-I-producing cell types from pDCs to conventional dendritic cells (cDCs) and macrophages including CD169+ splenic marginal zone macrophages [24, 26, 27]. To determine the source of the second wave of IFN-I during acute LCMV infection, we analyzed the expression of *Ifnb1* in different cell subsets by qPCR at day 5 p.i. (Fig. 2a). Ifnb1 expression was significantly upregulated in macrophages but not cDCs in acute infected mice. Only basal levels of Ifnb1 were observed in macrophages from chronic infected mice (Fig. 2b). Since the IFN-I response in antigen-presenting cells including macrophages has to be balanced by negative feedback regulation for enforced virus replication and efficient activation of the adaptive immune response [28], we tested for the presence of IFN-I-induced antiviral *Mx1* and the IFN-I receptor signaling suppressor *Usp18*. Acute infected mice showed elevated levels of *Mx1* and a concomitant upregulation of *Usp18* in macrophages (Fig. 2c). Conversely, macrophages from chronically infected mice showed only basal *Mx1* or *Usp18* expression (Fig. 2c). Thus, splenic macrophages of acute infected mice have a balanced IFN-I signature. To further define the macrophage subset that produces the second IFN-I wave, splenic CD169+ marginal zone macrophages from acute and chronic infected mice were enriched by magnetic beads and sorted by flow cytometry. Levels of *Ifnb1*, *Mx1* and *Usp18* transcripts were higher in cells from acute infected mice than in cells from chronic infected mice (Fig. 2d and Supplementary Fig. S4a), suggesting that CD169+ macrophages are the source of the second IFN-I wave. To further confirm this, acute infected CD169-DTR transgenic mice were treated with diphtheria toxin (DT) at day 3 p.i. to deplete CD169+ macrophages (Fig. 2e, f and Supplementary Fig. S4f). This significantly decreased the IFNB1 serum levels at day 5 p.i. (Fig. 2g). Together this demonstrates that CD169+ macrophages are the main producers of the second IFN-I wave in acute LCMV infection.

### Early CD169+ marginal zone macrophage depletion in chronic infection is mediated by CD8+ T cells

**Fig. 3:**
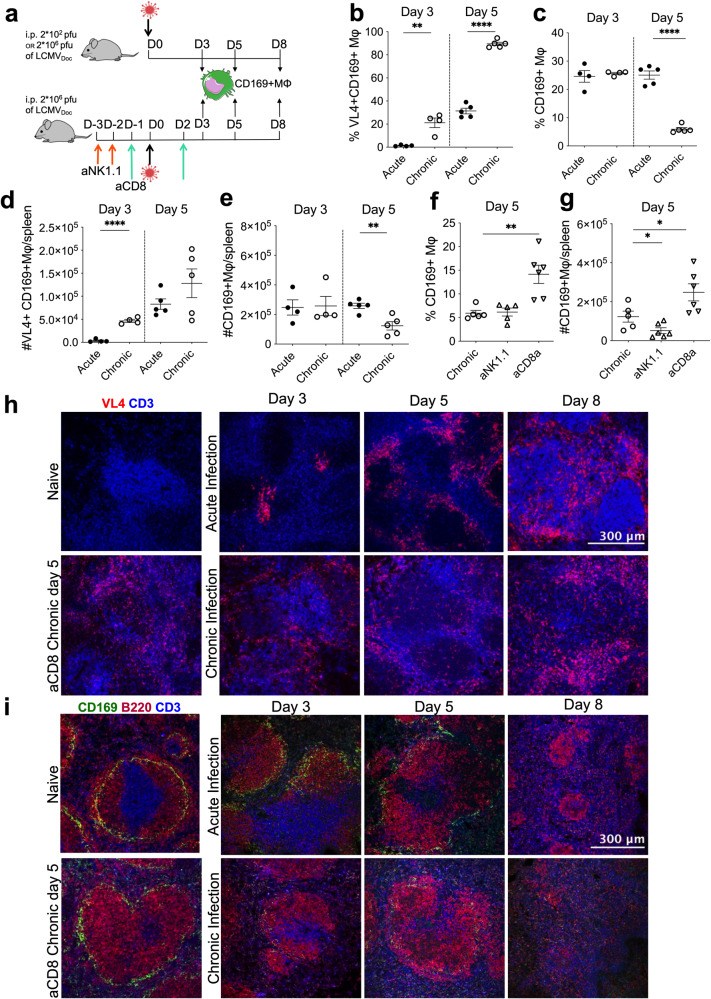
Temporal changes of the splenic marginal zone in acute versus chronic LCMV infection. **a** Schematic representation of timepoints of spleen sampling in acute and chronic LCMV infected mice and of anti-CD8α or anti-NK1.1 treatments in chronically LCMV-infected C57BL/6J mice. Frequencies of splenic LCMV-infected (VL4+) CD169+ macrophages (**b**) and of total splenic CD169+ macrophages (**c**) obtained by flow cytometry at days 3 and 5 post-infection from acute and chronic LCMV-infected mice. Absolute numbers of LCMV-infected (VL4+) CD169+ macrophages (**d**) and of total CD169+ macrophages (**e**) from days 3 and 5 post acute and chronic LCMV-infected mice. Frequencies (**f**) and absolute numbers (**g**) of splenic CD169+ macrophages at day 5 post-chronic LCMV infection in untreated and antibody-treated (anti-CD8α or anti-NK1.1) mice. For each group and time-point the mean ± SEM is shown; significance is determined via unpaired two-tailed t-test; *p ≤ 0.05; **p ≤ 0.01; ****p ≤ 0.0001. Immunofluorescence of spleen sections at the indicated time points after LCMV-infection from untreated or anti-CD8α antibody (aCD8) treated mice. Spleen sections were stained for VL4 (LCMV NP; red) and CD3 (T cells; blue) (**h**) and for CD169 (CD169+ Macrophages; green), CD3 (T cells; blue) and B220 (B cells; red) (**i**). One representative section of 3 mice per group is shown. Images were taken at 20X magnification, the scale bar indicates 300 µm.

Splenic marginal zone cells are known to be depleted during an acute LCMV infection [29]. Since the accumulated virus load at early time points during a chronic infection is increased over an acute infection [21], we hypothesized that this may translate to an earlier elimination of IFN-I-producing CD169+ marginal zone macrophages. To test this, LCMV-infected CD169+ macrophages and total CD169+ macrophages from spleens of acute and chronic infected mice at days 3 and 5 p.i. were quantified by flow cytometry (Fig. 3a–e). Frequencies of LCMV-NP-positive CD169+ macrophages increased to about 30% in acute infected mice and to about 90% in chronic infected mice from d3 to d5 p.i., respectively (Fig. 3b). The higher infection frequency was directly linked to the loss of this cell population in chronic infection (Fig. 3c), regardless of the size of the spleen that was increased at this time point (splenomegaly; unpublished observation). Next, to assess the cause of CD169+ macrophage loss, we depleted CD8+ cytotoxic T cells or NK cells which are known to be the two main cell types killing LCMV-infected cells in vivo [30, 31] and which appear earlier in chronic LCMV infection than in acute infection [2, 32]. For this, we treated mice with specific antibodies directed against NK cells or CD8+ T cells as outlined in Fig. 3a. Anti-CD8α antibody but not anti-NK1.1 antibody treatment prevented the loss of CD169+ marginal zone macrophages (Fig. 3f, g), demonstrating that CD8+ T cells are the culprit. In contrast, anti-NK1.1 antibody treatment further reduced the absolute number of CD169+ macrophages suggesting that NK cells did not play a significant role in macrophage killing (Fig. 3g). Moreover, T cells were increased after NK cell depletion (Supplementary Fig. S4e), confirming earlier observations and the role of NK cells in limiting CD8+ T cell-mediated lymphatic tissue damage [33].

To visualize LCMV distribution, splenic CD169+ marginal zone macrophage destruction within the lymphatic tissue, and their rescue by CD8+ T cell depletion, spleens of acute and chronic infected mice with and without anti-CD8α antibody treatment were analyzed by fluorescence microscopy at days 3, 5 and 8 (Fig. 3h, i). LCMV was detected in the tissue sections with the NP-specific VL4 antibody. At d3 p.i., LCMV was still confined to specific loci in acute infection while it was already spread throughout the marginal zone in chronic infection (Fig. 3h). This increased early LCMV dissemination in chronic infection was reflected in an earlier destruction of CD169+ splenic marginal zone macrophages which were already absent at d3 while still present at d3 and d5 but absent at d8 in acute infection (Fig. 3i). Depletion of the CD169+ macrophages was inhibited by anti-CD8α antibody treatment (Fig. 3i) demonstrating the importance of CD8+ T cells in CD169+ macrophage elimination. Thus, the splenic architecture of acute and chronic infected mice is disrupted with a different kinetics.

### The second wave of type I IFN in acute infected mice is required to induce inflammatory monocytes/macrophages and virus-specific CD8 T cells

**Fig. 4:**
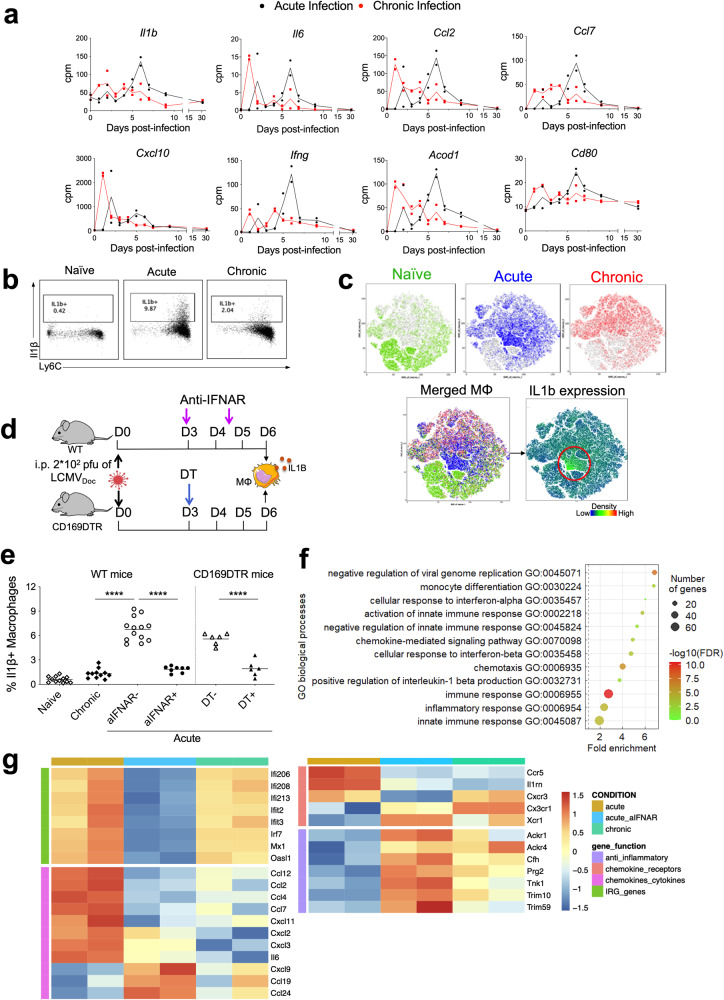
Characterization of the type I IFN-dependent inflammatory macrophage response in acute LCMV infection. **a** RNAseq-derived expression kinetics of genes related to pro-inflammatory macrophages in spleens from acute (black lines) and chronic (red lines) infected mice. **b** Representative flow cytometry plots showing IL1β-producing Ly6Chi macrophages. **c** T-distributed stochastic neighbor-embedding (tSNE) representation of macrophages from naïve, acute and chronic infected mice at day 6 post-infection. Distribution and relative expression of IL1β is shown in the merged plot. **d** Schematic representation of anti-IFNAR antibody and diphtheria toxin (DT) treatments in acutely LCMV-infected WT and CD169DTR mice, respectively. **e** Percentages of IL1β-producing macrophages in the spleen at d0 (naïve) or d6 post-acute or chronic infection in untreated (aIFNAR−/DT−) or treated (aIFNAR+/DT+) mice. For each group, the mean of *n* = 6 to 13 mice, representative of two independent experiments, is shown. ****p ≤ 0.0001; unpaired two-tailed t-test. **f** Enriched GO terms (obtained from DAVID) on genes differentially expressed in spleens of aIFNAR-treated versus untreated mice at day 6 post-acute LCMV infection. **g** Heatmap illustrating the relative expression of genes in spleens from acute, acute aIFNAR-treated and chronic-infected mice at day 6 post-infection.

Co-expressed genes within WGCNA-derived modules belong to functionally related biological processes [34]. To determine the functional role of the second IFN-I wave in acute infection, we therefore analyzed the genes that are coexpressed with the IFN-I genes of the acute-brown module. Interestingly, there were several genes associated with inflammation that were not coexpressed with IFN-I genes of the chronic-brown module during chronic infection (Fig. 4a). This suggested that the second IFN-I wave is specifically linked to an inflammatory response that occurs during acute infection. Indeed, our previous work demonstrated that monocytes/macrophages from day 6 post-acute LCMV infection but not from chronic infection displayed a pro-inflammatory profile [21]. In line with this, flow cytometry analysis (see Supplementary Fig. S5) demonstrated the induction of IL1β-producing macrophages at day 6 after acute LCMV infection that was significantly reduced in chronic infection (Fig. 4b, d). This difference in macrophage phenotypes can be visualized by t-Distributed Stochastic Neighbour Embedding (t-SNE) analysis that reveals IL1β+ expression as a differentiating feature between acute and chronic infection (Fig. 4c). To investigate whether the second wave of IFN-I expression is required for the induction of this pro-inflammatory response, we abrogated IFN-I signaling by anti-type I IFN receptor (aIFNAR) treatment in WT C57BL/6J mice or depleted IFN-I-producing CD169+ macrophages by diphtheria toxin (DT) treatment in CD169-DTR transgenic mice [35], respectively (Fig. 4d). Both treatments resulted in a significant reduction of IL1β-producing macrophages at day 6 p.i. (Fig. 4e).

To further characterize the spectrum of cytokines, beyond IL1β, affected by the second wave of IFN-I signaling, mRNA from spleen of acute LCMV-infected mice with or without IFNAR blockage and of chronic LCMV-infected mice at day 6 p.i. were isolated and transcriptomes determined by RNAseq. DE genes from the transcriptomes of aIFNAR-treated and untreated acute infected mice were enriched in the GO terms “monocyte differentiation”, “chemokine-mediated signaling pathway”, “chemotaxis”, and “inflammatory response”, thus highlighting the role of the second IFN-I wave in modulating the inflammatory environment (Fig. 4f). This becomes apparent in the heatmap of the most differentially expressed genes taken from the GO terms above (Fig. 4g). Genes related to chemokine receptors and proinflammatory cytokines/chemokines are upregulated in acute infected mice, while downregulated in aIFNAR-treated mice. Anti-inflammatory genes instead follow the opposite trend, they are downregulated in acute-infected mice and upregulated in aIFNAR-treated mice. Finally, aIFNAR-treated mice show a transcription pattern that is closer to chronic infected mice than to acute infected mice. Together this demonstrates that the second IFN-I wave produced by CD169+ marginal zone macrophages is required for the inflammatory macrophage response at d6 post acute infection, and lack of this, due to prior CD169+ macrophage destruction, results in the significantly reduced inflammatory response in chronic infection.

**Fig. 5:**
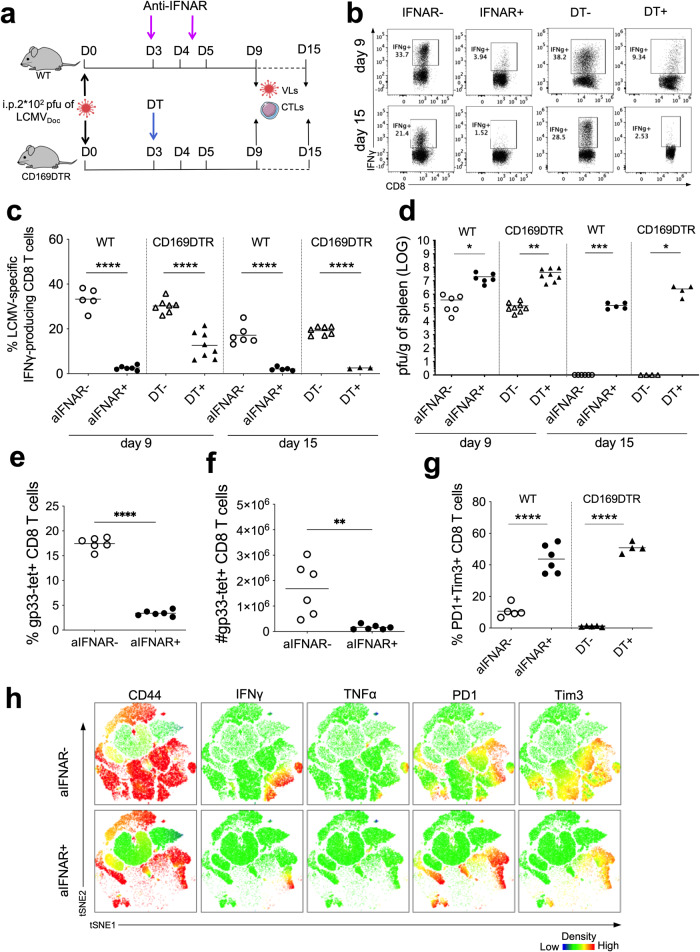
Characterization of type I IFN-dependent CTLs response in acute LCMV infection. **a** Schematic representation of anti-IFNAR antibody and diphtheria toxin (DT) treatments in acutely LCMV-infected WT and CD169DTR mice, respectively. **b** Representative flow cytometry plots showing gp33-responsive IFNγ-producing CD8+ T cells. Frequency of gp33-responsive IFNγ-producing CD8+ T cells (**c**) and viral loads (**d**) in WT mice untreated (aIFNAR−) or treated (aIFNAR+) with anti-IFNAR antibody, and in CD169DTR mice untreated (DT−) or treated (DT+) with diphtheria toxin (DT) at days 9 and 15 post-infection. Frequencies (**e**) and absolute numbers (**f**) of gp33-tet+ CD8+ T cells after anti-IFNAR antibody treatment at day 9 post-infection. **g** Frequency of Tim3+PD1+ exhausted CD8+ T cells in WT mice after anti-IFNAR antibody treatment, and in CD169DTR mice after DT treatment at day 9 post-infection. **h** tSNE representation of CD8 T cells from untreated (aIFNAR−) and treated (aIFNAR+) infected mice at day 9 post-infection. Distribution and relative expression of selected markers in T cells are shown. For each group, the mean of *n* = 4 to 8 mice, is shown. *p ≤ 0.05; **p ≤ 0.01; ***p ≤ 0.001; ****p ≤ 0.0001; unpaired two-tailed t-test.

IFNAR signaling in CD8 T cells is critical for the generation of effector and memory cells [36]. To investigate the influence of IFN-I produced by CD169+ marginal zone macrophages on the virus-specific CD8 T cell response, we blocked IFNAR signaling in acute infected C57BL/6J WT mice and depleted CD169+ macrophages in acute infected CD169DTR mice at days 3–5 p.i. (Fig. 5a). Both treatments resulted in (i) decreased functionality (Fig. 5b, c) and numbers of LCMV-specific CD8 T cells (Fig. 5e, f) at days 9 and 15 p.i., and (ii) increased virus titers (Fig. 5d). Importantly, mice lacking either IFNAR signaling of the second IFN-I wave or CD169+ macrophages, became chronically infected with LCMV as shown by viremia beyond d15 (Fig. 5d) and signs of CD8+ T cell exhaustion as evidenced by the increase of PD1+Tim3+ cells (Fig. 5g, h). Together these data highlight the impact of the CD169+ macrophage destruction kinetics on immune response regulation. In acute infection, the delayed CD8 T cell-mediated killing of CD169+ marginal zone macrophages is instrumental for the induction of a second IFN-I wave which coordinates an optimally-timed sequence of innate and adaptive immune events. In chronic infection, the early CD8 T cell-mediated killing of CD169+ marginal zone macrophages eliminates the primary source of the second IFN-I wave and thus is a key regulator for this infection outcome.

### Early IFN-I kinetics determines the appearance of fibrosis in lymphatic tissue

**Fig. 6:**
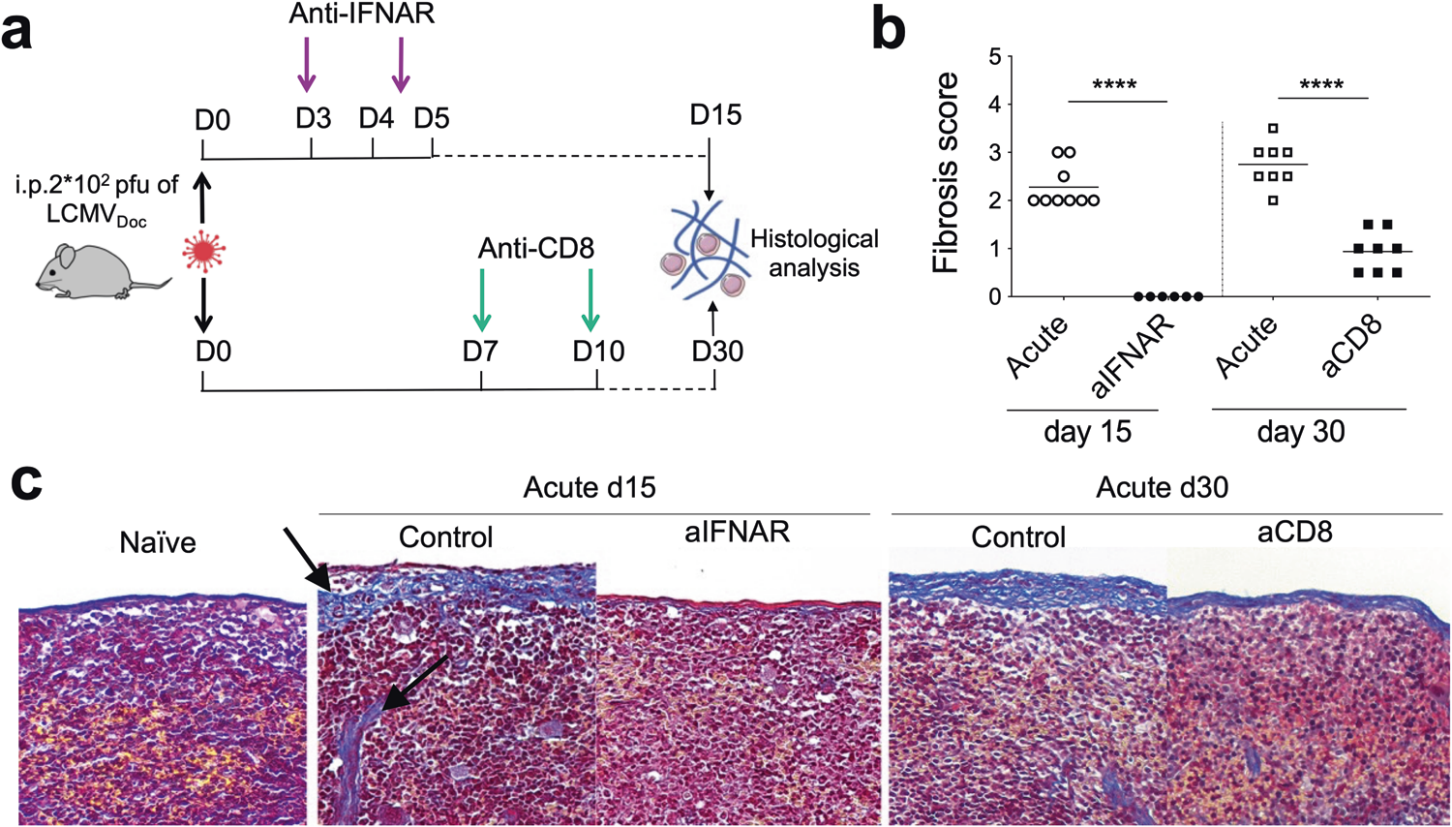
The resolution of LCMV acute infection is associated to CTL-mediated spleen fibrosis. **a** Schematic representation of anti-IFNAR (aIFNAR) and anti-CD8α (aCD8) antibody treatments in acutely LCMV-infected WT mice. Spleen fibrosis score (**b**) and representative images of Masson’s Trichrome Stained spleen sections (**c**) used to score the fibrosis in each group on days 15 and 30 post-infection; the black arrows indicate deposits of collagen fibers (in blue – Masson trichrome stain) characteristic of fibrosis. Data shown are the mean of *n* = 6 to 9 mice. ****p ≤ 0.0001; unpaired two-tailed t-test.

Lymphoid tissue fibrosis is a typical feature of chronic infections such as HIV and is intimately linked to chronic immune activation and inflammation within the affected tissues [37, 38]. In our previous study, we showed that also during acute infection, lymphoid tissue fibrosis occurs and persists even when the virus is already well-controlled [21]. Notably, acute LCMV infection induces a strong cytotoxic T-lymphocyte (CTL) response [39] which results in varying degrees of immunopathology during CTL-mediated killing of virus-infected cells [40]. However, no previous work ever characterized the histopathological costs of a well-coordinated immune response against LCMV. This prompted us to investigate the role of the IFN-I-mediated CTL response in the development of fibrosis during acute LCMV infection. For this we either blocked IFNAR signaling by anti-IFNAR antibodies or depleted CD8+ T cells at the peak of the CTL response [21] as depicted in Fig. 6a. Importantly, blockage of signaling of the second IFN-I wave entirely prevented spleen fibrosis at d15 p.i. (Fig. 6b, c). Similarly, anti-CD8 antibody treatment strongly reduced the occurrence of fibrosis at d30 p.i. These findings indicate that the generation of fibrotic tissue in spleens from acute infected mice is a consequence of the IFN-I-dependent antiviral CD8 T cell response that is required to resolve the infection. Taken together, our findings demonstrate that the significant immune-related tissue damage observed in mice after acute LCMV infection is the cost for effective virus control.

## Discussion

How early virus-host interaction events and their dynamics influence virus infection fates remains elusive. Here we demonstrate that acute and chronic LCMV infection outcomes are the result of a distinct kinetics of lymphatic tissue alterations that manifest themselves as differences in type I IFN responses. We unmask the spatio-temporal immune events that cause these differences and link the changes in the IFN-I response to subsequent characteristics of the different infection outcomes. Together, this illustrates the importance of a temporally well-coordinated sequence of innate and adaptive immune events for virus infection control.

Our systems-based approach to analyze acute and chronic LCMV infection fates in mice enabled mechanistic and conceptional advances that go beyond current knowledge. First, while IFN-I is a well-established major player in the early antiviral response against virus infection [41], we show here that acute and chronic LCMV infections fundamentally differ in their IFN-I responses: IFN-I genes are expressed in two waves in acute infection but have only one wave in chronic infection. Second, while it is known that CD169+ macrophages contribute to IFN-I production during LCMV infection [27], we show here that the IFN-I expression by splenic CD169+ macrophages during acute infection corresponds to the 2^nd^ IFN-I wave and has a polyfunctional role in bridging innate and adaptive immunity. Third, from the pioneer work of the *Zinkernagel* group, it has been widely accepted that CD8 T cells contribute to spleen architecture disruption during LCMV infections [42]. Here we describe that CD8 T cell-mediated killing of CD169+ macrophages in chronic infection dampens the production of the second wave of IFN-I. This results in the appearance of chronic infection-specific immune features such as the lack of inflammatory macrophages and the generation of exhausted T cells. Forth, we previously showed that LCMV infection can induce lymphatic tissue fibrosis [21]. Fibrosis is a common feature also in human infections like those with HIV-1 and is associated with a drop in immune functioning [43]. Here we demonstrate that spleen fibrosis in LCMV acute-infected mice is a consequence of the IFN-I-dependent antiviral CD8 T cell response that is required to resolve the infection. Thus, fibrosis is ‘the price’ the host has to pay for effective virus control.

The crosstalk of viruses with the IFN-I response of the host organism can determine infection outcomes. Many viruses encode IFN antagonists that suppress or delay IFN-I expression and provide viruses with a kinetic advantage over host-mediated restriction mechanisms [44, 45]. In case virus expansion and restriction mechanisms become very unbalanced, virus infection may be highly pathogenic. Remarkable examples of this are infections with the 1997 Hongkong Flu strain that carried a potent IFN antagonistic NS1 protein [46] or infections of host organisms that have weakened IFN-I responses due to autoantibodies [47] or genome variation within IFN-inducible restriction factors [48]. However, in our model system of acute and chronic LCMV infections, the same LCMV strain was used and thus, different antagonistic viral protein functions were not operative. Rather it was the system’s intrinsic kinetics in the destruction of infected marginal zone CD169+ macrophages by CD8 T cells that caused a differential IFN-I expression. These cells were the dominant source of the second IFN-I wave at day 5 in low-dose acute infection, while in high-dose chronic infection CD169+ macrophages were already disappearing by day 3. Thus, the 2-wave behavior of IFN-I during acute infection is a reflection of IFN-I production mediated by different cell subsets at different timepoints, first from pDCs, then from cDCs and macrophages [27]. Hence, the subsequent IFN-I-dependent immune components characterized in acute infection, namely proinflammatory macrophages and effector CD8 T cells, did not arise in chronic infection [21]. Importantly, the loss of the splenic marginal zone was not a specific event during chronic infection, since it also happened in acutely infected mice albeit at later times. Indeed, this differential kinetics of spleen disruption perfectly fits to the described earlier appearance and expansion of CTLs in chronic LCMV Doc-infected mice [2]. Together this indicates that the decision point for the infection fate was entirely determined by a kinetic feature of the virus-induced immune response.

CD169+ marginal zone macrophages play a central role in coordinating the early immune events required to control a virus infection. They are crucial for the filtering and retention of pathogens from blood [49], participate in the distribution and presentation of antigens to B cells [50], collaborate with cross-presenting DCs in the activation of CD8 T cells [51] and produce IFN-I when productively infected with viruses such as Herpes Simplex Virus or LCMV [27, 52]. The prompt disappearance of CD169+ macrophages and subsequent lack of IFN-I production observed in chronic LCMV-infected mice represents the loss of a critical component of the coordinated antiviral immune response. Indeed, the disruption of this regulatory mechanism during acute infection through IFNAR blockage or the early depletion of CD169+ macrophages in CD169DTR mice resulted in the induction of exhausted T cells and the subsequent development of a chronic infection. Together, our results further highlight the essential role of marginal zone macrophages in coordinating innate and adaptive immune responses for efficient virus control.

We have previously shown that acutely LCMV-infected mice develop spleen fibrosis which is maintained even when the virus is already well controlled [21]. Here we demonstrate that these histological changes are a consequence of the killing of infected cells by cytotoxic CD8 T cells, which in turn are induced by the second wave of IFN-I production by CD169+ macrophages. Thus, lymphoid tissue fibrosis is ‘the price’ the host has to pay for the successful clearance of the virus. However, to what extent fibrosis induced after acute virus infection affects organ functionality and therefore the capacity of the immune system to properly respond to subsequent challenges has still to be determined. Previous studies have shown that fibrosis footprints in lymphatic tissues derived from infections are linked to a significant impairment of responses towards vaccines [53] and subsequent other infections [54]. Moreover, lymphatic tissue fibrosis in chronic HIV and SIV infections impairs immune cell interactions [43, 55–57]. Nonetheless, fibrosis during acute or chronic virus infections develops in the distinct physiological contexts of either fully functional or immune-compromised environments, respectively, and thus may differ in their immune modulating properties.

In conclusion, our systems immunology-based analysis of acute and chronic LCMV infections identifies a missing regulatory element in the coordination of systemic virus-host interactions: the induction of a second IFN-I peak in acute infection and its lack in chronic infection. This helps to better appreciate the complexity of the tightly regulated IFN-I responses early during a virus infection, and reveals several IFN-I-dependent immune events that influence infection fates. Moreover, the systemic approach used illustrates the importance of analyzing immune responses as a whole, considering the relationship among all its components. Only by integrating “numbers game” and “immune geography”, i.e. the kinetics of virus replication and immune responses at spatial resolution, will we fully understand virus-host battles and their outcomes [14]. Finally, our study not only provides a unique IRG transcriptome data set during acute and chronic virus infections but also suggests directions for immunotherapeutic strategies to avoid chronicity. An inspection of the results in human infections is clearly indicated.

## Methods

### Mice

C57BL/6J mice (RRID:IMSR_JAX:000664) were purchased from Charles River Laboratories and CD169-DTR transgenic mice, generated in the RIKEN BRC laboratory (Dr. Kenji Kohno and Dr. Masato Tanaka) through the National BioResource Project of the MEXT/AMED (Japan), were a kind gift from Andres Hidalgo (Madrid, Spain). All mice were bred and maintained under specific pathogen-free conditions at in-house facilities. The number of animals for each experiment was determined based on previous experience with the model system.

### Virus infections

LCMV strain Docile (LCMV_Doc_) was used for mouse infections. The virus was grown, stored and quantified according to previously published methods [21]. Mice were infected by intraperitoneal (i.p.) injection of either 2 ×10^2^ or 2 ×10^6^ plaque-forming units (pfu) of LCMV_DOC_ to respectively induce an acute or a chronic infection.

### In vivo treatments with antibodies

To block the IFN-I signaling at the desired timepoint, C57BL/6J mice were intraperitoneally (i.p.) inoculated with 500 µg of anti-IFNAR (aIFNAR) antibody (clone MAR1-5A3; Leinco Technologies) or 500 µg of a mouse IgG1 isotype control (clone MOPC21; Bio X Cell) on days 3 and 4.5 post-infection. In order to block the proinflammatory response at day 6 p.i.

### In vivo cell depletion

In order to deplete CD169+ macrophages at day 5 p.i., diphtheria toxin (DT; Sigma-Aldrich) was i.p. injected into CD169DTR transgenic mice (30 μg/kg) at day 3 post-acute infection. Physiological serum was used as a control vehicle. CD8 T cell depletion in acute and chronic LCMV infection was performed by i.p. administration of 200 μg of anti-CD8α antibody (clone 2.43; Bio X Cell) respectively at days -1 and 2 p.i. and at days 7 and 10 p.i. For depletion of NK cells, chronic LCMV-infected mice were i.p. injected with 75 μg of anti-NK1.1 antibody (clone PK136; Bio X Cell) at days -3 and -2 p.i.

### Flow cytometry and sorting

Spleens were processed into a single-cell suspension and stained as previously described [21]. For staining and sorting of CD169+ macrophages, spleens were previously enzymatically digested with a mix of Liberase-DNAse (0.16 mg/ml; 0.2 mg/ml) (Roche) for 30 min. Splenocytes were stained for viability with either Live/Dead Fixable Violet Cell Stain (ThermoFisher Scientific) or Fixable Viability Stain 620 (BD biosciences) or Fixable Viability Stain 780 (BD biosciences). After washing, cells were incubated with Fc block (BD Biosciences) to block non-antigen-specific binding of immunoglobulins to Fc receptors and then stained with fluorochrome-labeled antibodies targeting surface or intracellular proteins. For gp33-tetramer stainings, biotinylated MHC class I monomer (MBL International) was tetramerized by adding PE-conjugated Streptavidin over 10-time intervals and then added together with extracellular antibodies. For detection of IFNɣ-producing T cells, intracellular cytokine staining was performed after 5 h ex vivo stimulation with GP_33_ (1 μg/ml) in the presence of Brefeldin A (BFA, Sigma). To detect IL-1b-producing macrophages, spleens were harvested in media containing BFA and antibody staining was performed without additional stimulation. After surface antibody staining, cells were fixed with 2% Formaldehyde, permeabilized in Perm/Wash buffer (PBS 1% FCS, NaN_3_ 0,1%, Saponin 0,1%) and then stained with intracellular antibodies for IFNɣ, IL-1b and VL-4. Flow cytometry samples were acquired on a SP6800 Spectral (Sony) or an Aurora (Cytek) analyzer. FACS data were analyzed using FlowJo 10.1 software (Tree Star Inc). FACSAria II Sorter (BD Biosciences) was used to sort cells in RLT buffer (Qiagen) with sorting purity >95%.

### Magnetic-activated cell sorting

To sort splenic CD169+ macrophages, mice were injected with a biotin-anti-CD169 antibody (36 μg; Abcam) and 10 min later, spleens were harvested and digested with a Liberase-DNAse mix. CD169+ cells were stained with anti-biotin microbeads (Miltenyi) for 30 min and purified by magnetic-activated cell sorting using LS columns (Miltenyi). CD169+ cells were additionally sorted by flow cytometry after staining with streptavidin-phycoerythrin (Thermo Fisher) and CD11b. Sorted cells were collected in RLT buffer (Qiagen) and kept on ice. Sorting purity was >95% for all populations.

### Histological staining

Spleen samples were fixed overnight with 4% buffered formaldehyde and paraffin-embedded. For semi quantification of fibrosis, Masson’s Trichrome was performed on 3 µm thick spleen cuts that revealed collagen fibers in blue. A set of values was defined from 0 to 10: a score of 0 represents normality; a score of 10 would correspond to a spleen in which the parenchyma has totally been replaced by connective tissue.

### Immunofluorescence

Freshly removed spleens were fixed for 24 h in Cytofix buffer (BD), and dehydrated for 12 h in 30% sucrose at 4 °C. Next, samples were OCT-embedded (Tissue-TEK) in cryomolds and stored at −80 °C before proceeding with sectioning. Serial tissue sections of 10 µm in thickness were obtained using Leica Cryostat and mounted on Superfrost Plus slides (Fisher Scientific) and frozen at −80 °C. Spleen sections were rehydrated in 0.1 M Tris buffer (1 M Tris powder in deionized water), blocked for 2 h in standard blocking buffer (1% Bovine serum albumin, 0.3% Triton 100X in 0.1 M Tris buffer; Sigma-Aldrich) and incubated overnight at 4 °C with primary and/or conjugated antibodies. CD169+ splenic marginal zone macrophages were visualized by staining with CD169 AF647 (MOMA-1; clone 3D6.112; Biolegend). B cells and T cells were identified using antibodies against B220 (rat anti-mouse clone RA3-6B2; Biolegend) and CD3 (hamster anti-mouse clone 145-2C11; Biolegend). For unconjugated primary antibodies, species-specific secondary antibody coupled to Alexa Fluor 488 (goat anti-hamster; Biolegend), or Alexa Fluor 555 (goat anti-rat; Thermofisher) fluorochromes were incubated for 8 h at 4 °C. Sections were washed, DAPI-stained (4’,6’-diamino-2-phenylindoledihydrochloride; Sigma) to visualize nuclei and finally mounted using Vectashield mounting media (Vector laboratories). Images were captured using a Leica SP5 Inverted Confocal microscope and analyzed with ImageJ software.

### Virus load quantification

Viral titers from spleens of infected mice were determined by focus forming assay as previously described [58]. Briefly, spleens were frozen at −80 °C right after collection. Tissue was mechanically disrupted and resuspended in 500 μl of DMEM 2%FBS. One in ten-fold dilutions were prepared and overlaid onto MC57 cell monolayers in 24-well plates. After 48 h of incubation at 37 °C 5% CO_2_, staining was performed using monoclonal rat anti-LCMV antibody (VL-4) for 1 h, followed by Peroxidase Anti-Rat IgG Polyclonal Ab (Jackson ImmunoResearch). Plaques were visualized using DAB Peroxidase substrate kit (Vector Laboratories).

### ELISA

Mouse blood was collected from the submandibular vein and clotted at room temperature for 20 min before centrifugation at 2000 × *g* for 10 min. The serum was collected and stored at −80 °C. VeriKine-HS Mouse Interferon-Beta ELISA Kit (Pbl Assay Science) was used to determine IFNB1 levels in serum, according to the manufacturer’s instructions.

### Quantitative real-time PCR

Total RNA was extracted from whole spleens or from sorted cells, using respectively Qiagen RNeasy Mini Kit and Qiagen RNeasy Micro Kit. Quality and concentration of RNA were determined by an Agilent Bioanalyzer. RNA from whole spleens was reverse-transcribed to cDNA using SuperScript III Reverse Transcriptase (ThermoFisher). Quantitative real-time PCR (qPCR) was then performed using SYBR select master mix (ThermoFisher) via the Quantstudio 12 K flex (ThermoFisher). RNA from sorted cells, was directly used to perform qPCR with a one-step Quantitect SYBR green kit (Qiagen) via the Lightcycler 480 II (Roche) according to manufacturer’s instructions. Primers for all genes were designed using the program Primer Express 3.0 (Applied Biosystems) and ordered from Biomers. Gene expression was normalized to that of *Gapdh* and compared for the study groups.

### RNA-seq library preparation, sequencing and bioinformatic analysis

Total RNA from spleens was isolated from two mice/ group/ time point and submitted for sequencing to the Genomics Unit of the Centre for Genomic Regulation (CRG, PRBB). Sequencing libraries were obtained after removing ribosomal RNA by a Ribo-Zero kit (Illumina). cDNA was synthesized and tagged by addition of barcoded Truseq adapters. Libraries were quantified using the KAPA Library Quantification Kit (KapaBiosystems) prior to amplification with Illumina’s cBot. Four libraries were pooled and sequenced (single strand, 50 nts) on an Illumina HiSeq2000 sequencer to obtain 50–60 million reads per sample. Bioinformatic analysis, carried out according to previously published methods [21]. The total number of mapped genes used to perform further analysis was similar at all time points.

### Quantification and statistical analysis

Graphs were compiled and statistical analyses were performed by Prism (GraphPad software). Statistical significance was evaluated with the unpaired t-test when comparing two groups and one-way ANOVA multiple comparison test when comparing more than two groups. Non-significant differences were indicated as “ns”. *P*-values below 0.05 were considered significant and were indicated by asterisks: *p < 0.05; **p < 0.01; ***p < 0.001; ****p < 0.0001.

### Reporting summary

Further information on research design is available in the Nature Research Reporting Summary linked to this article.

### Supplementary information

Supplemental figures

Reporting Summary

## Data Availability

All the data and information concerning this study will be made available upon request.
