# Supplementary material for: Differential kinetics of splenic CD169+ macrophage death is one underlying cause of virus infection fate regulation
Source: Cell Death Dis. 2023 Dec 18;14(12):838. doi: 10.1038/s41419-023-06374-y (PMC10728219; doi:10.1038/s41419-023-06374-y)
Supplement: Supplementary file 1 — Supplemental figures [file 41419_2023_6374_MOESM1_ESM.docx]

# Supplementary figure legends

**Fig.S1:** (a) WGCNA and (b) identification of modules of coregulated genes from acute and chronic LCMV infections.

**Fig.S2:** Enriched GO terms (obtained from DAVID) for genes of acute-brown and chronic-brown modules.

**Fig.S3:** (a) Expression kinetics of selected IRGs (*Irf7*, *Stat2*, *Mx1* and *Oasl1*) and (b) qPCR of *Mx1* from spleens of acute- and chronic-infected mice at the indicated time points.

**Fig.S4:** (a) *Mx1* and *Usp18* gene expression levels measured by qPCR using RNA extracted from sorted CD169+ macrophages from acute- and chronic-infected mice. (b) RNAseq-derived expression kinetics of *Siglec1* (CD169+ Macrophage marker) in spleens from acute (black lines) and chronic (red lines) infected mice. Significance of the difference at day 5 was assessed by an unpaired two-tailed t-test. P-values below 0.05 were considered significant and were indicated with asterisks: *p< 0.05.

(c) Percentages of macrophages in spleens obtained by flow cytometry at day 5 post-infection. (d-e) Quantification of NK cells (d) and T cells (e) at day 5 p.i. in aNK1.1-treated and untreated chronic infected mice. (f) Quantification of CD169+ Macrophages in DT-treated and untreated acute-infected CD169DTR mice.

**Fig.S5:** Flow cytometry gating strategy to define Il1β-producing macrophages.

# Supplementary figures

## Fig.S1:


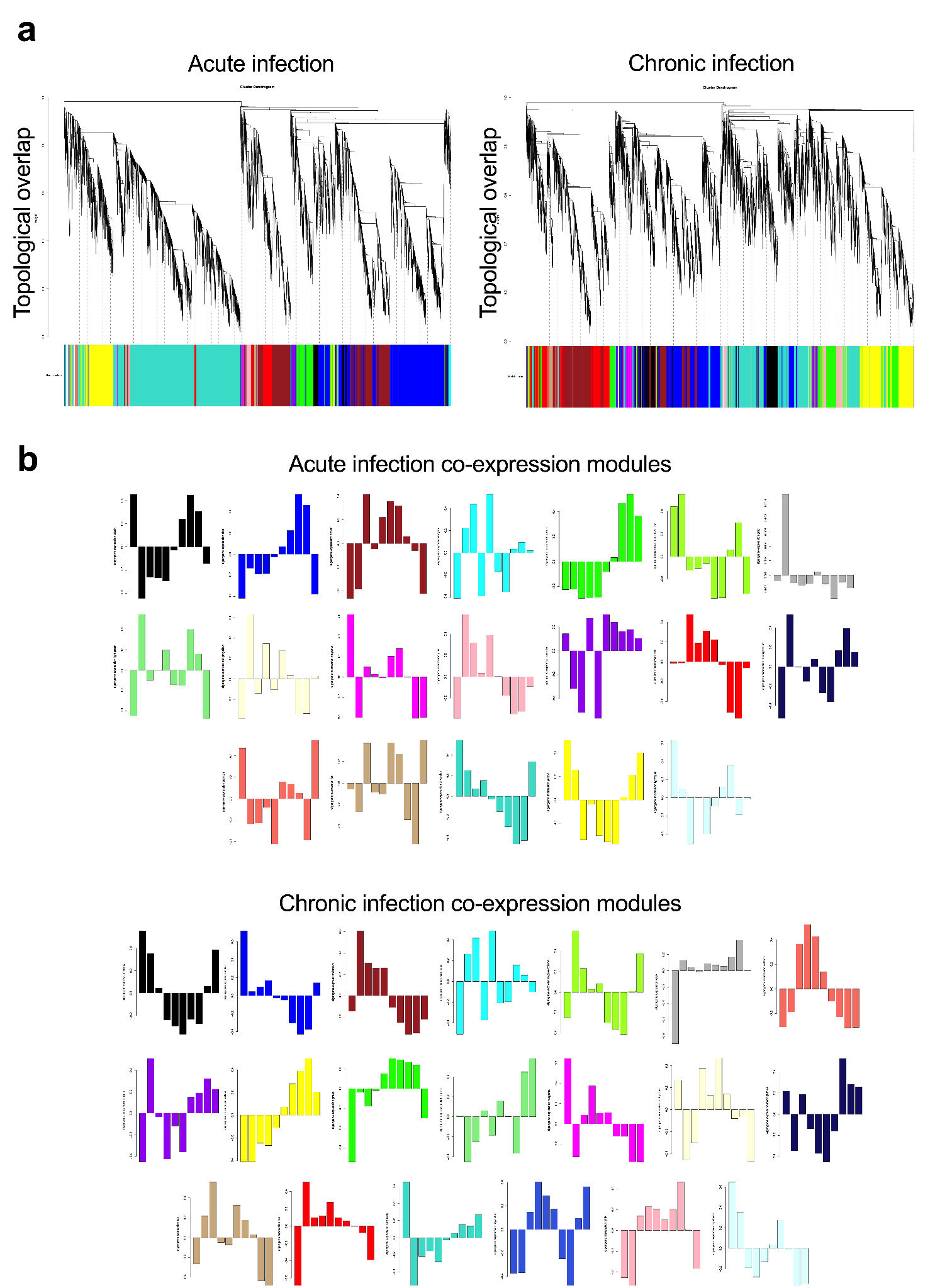


## Fig.S2:


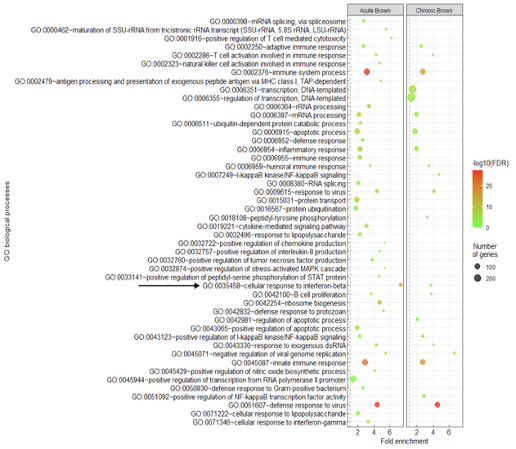


## Fig.S3:

**
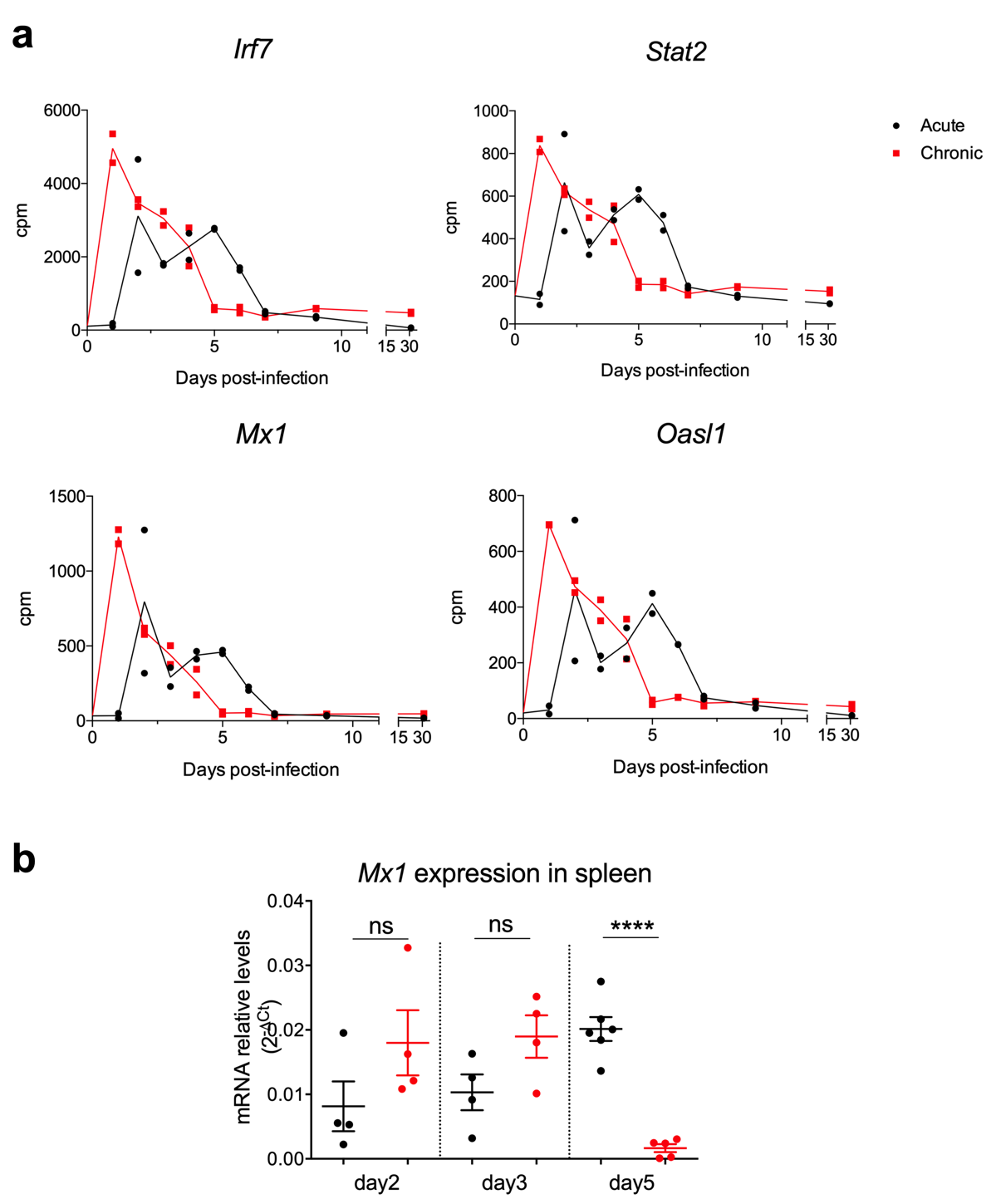
**

## Fig.S4:

##
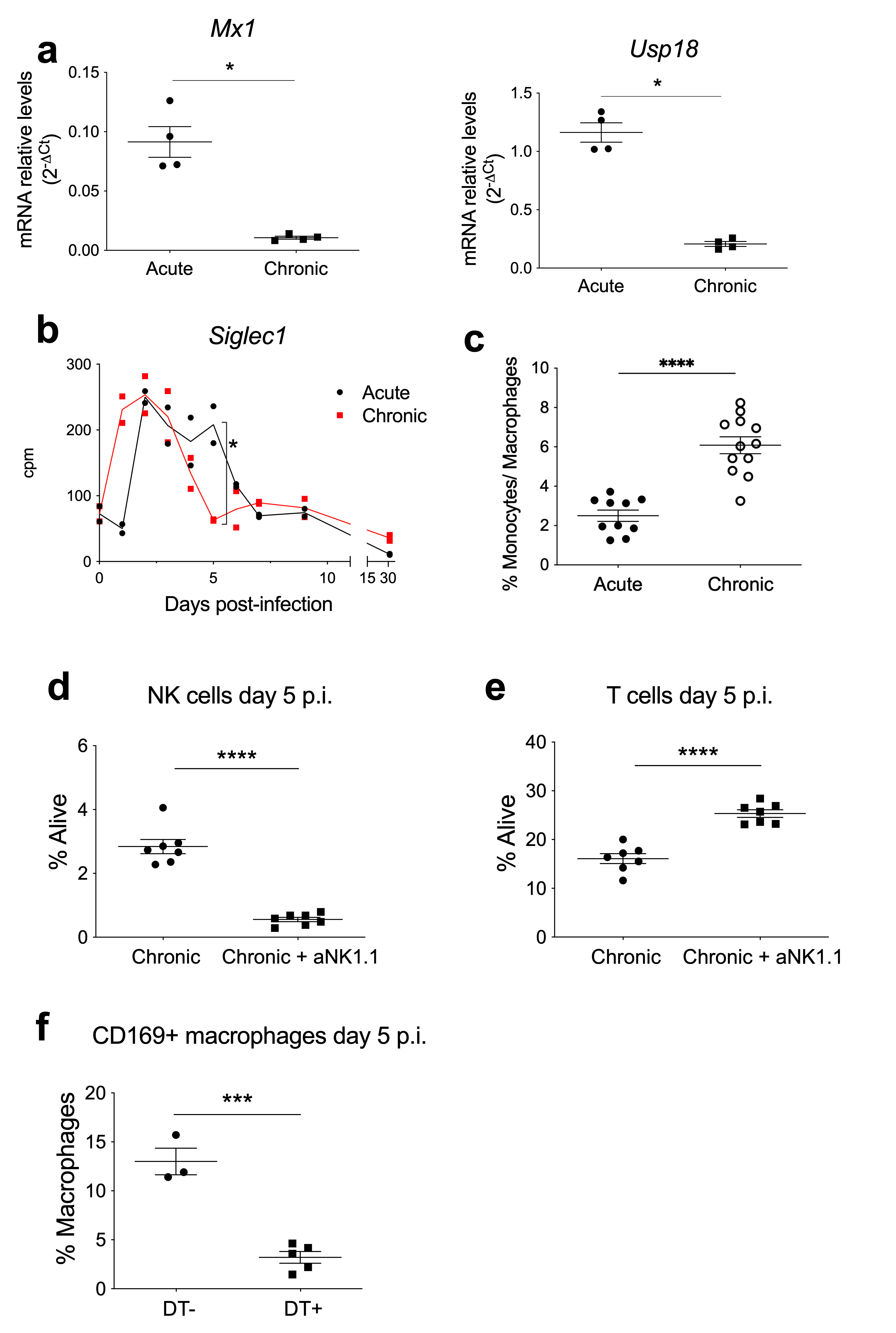


## Fig.S5:

**
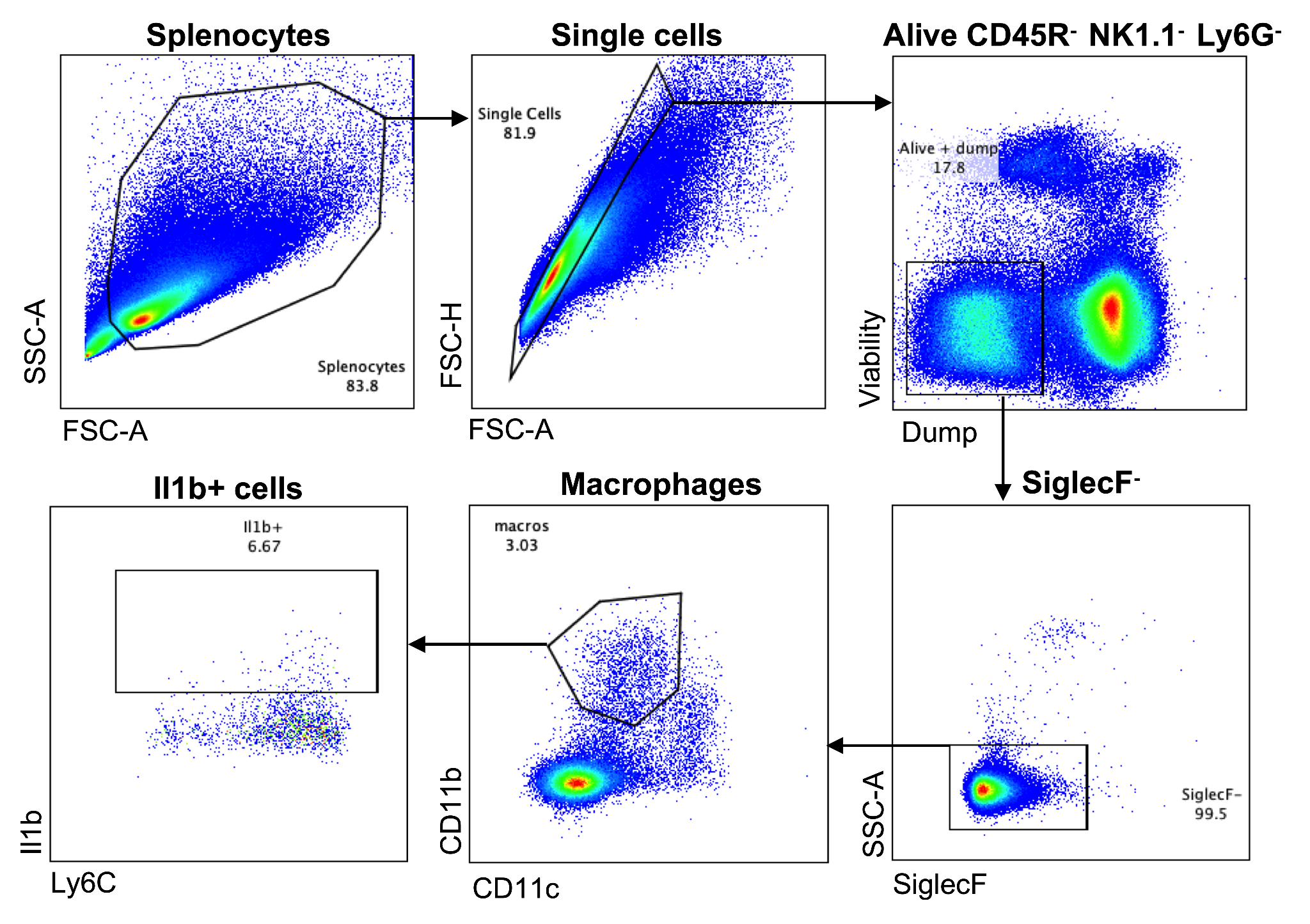
**
